# Aquaporin-4 and MicroRNA Expression in Meningiomas: A Tissue-Level Exploratory Analysis

**DOI:** 10.3390/biomedicines14051125

**Published:** 2026-05-15

**Authors:** Huseyin Omer Keskin, Emre Ozkara, Ebru Erzurumluoglu, Zuhtu Ozbek, Evrim Yilmaz, Funda Canaz, Didem Arslantas, Sevilhan Artan, Ali Arslantas

**Affiliations:** 1 Department of Neurosurgery, Faculty of Medicine, Eskisehir Osmangazi University, 26000 Eskisehir, Turkey; hsynomer@gmail.com (H.O.K.);; 2 Department of Genetics, Faculty of Medicine, Eskisehir Osmangazi University, 26000 Eskisehir, Turkey; 3 Department of Pathology, Faculty of Medicine, Eskisehir Osmangazi University, 26000 Eskisehir, Turkey; 4 Department of Public Health, Faculty of Medicine, Eskisehir Osmangazi University, 26000 Eskisehir, Turkey

**Keywords:** meningioma, aquaporin-4, microRNA, miR-216a, miR-320a, LINC00461, molecular pathology, tumor heterogeneity

## Abstract

**Background:** Meningiomas exhibit considerable biological heterogeneity that is not fully captured by histopathological grading. Tissue-based molecular markers may provide complementary insight into tumor biology within routine diagnostic settings. **Methods:** Formalin-fixed paraffin-embedded tissue samples from 65 intracranial meningiomas and 13 non-neoplastic controls were analyzed. Aquaporin-4 (AQP4) expression was assessed using immunohistochemistry, while miR-216a, miR-320a, and LINC00461 levels were quantified by means of RT-qPCR. Expression patterns were compared across groups and evaluated in relation to histological grade. **Results:** AQP4 expression was significantly reduced in meningiomas compared with controls and showed a further decrease in higher-grade tumors. Although expression of miR-216a and miR-320a was also lower in tumor samples, these differences did not reach statistical significance. Correlation analysis revealed modest but significant associations between AQP4 and miR-216a, as well as between miR-216a and miR-320a. Individual markers demonstrated limited discriminatory performance; however, combined expression patterns suggested underlying molecular variability across tumor grades. **Conclusions:** Our findings indicate that AQP4 downregulation represents a consistent feature in meningiomas, while associated microRNA alterations may reflect coordinated but context-dependent expression patterns. Although these markers are not sufficient as standalone diagnostic tools, their combined tissue-level assessment may provide complementary information on tumor heterogeneity. These findings should be interpreted as exploratory and highlight the need for further validation in larger and mechanistic studies.

## 1. Introduction

Meningiomas account for nearly one third of all primary intracranial tumors and represent the most common extra-axial neoplasm of the central nervous system [1,2]. Although the majority are classified as World Health Organization (WHO) grade 1 and exhibit relatively benign clinical behavior, a subset demonstrates recurrence, aggressive growth, and resistance to treatment that cannot be fully predicted by histopathological grading alone [3,4].

The 2021 WHO classification emphasizes the integration of molecular and genetic alterations into tumor diagnosis and grading to improve prognostic accuracy beyond conventional morphology [5,6]. In meningiomas, molecularly integrated grading systems and DNA methylation-based classifications have shown that tumors with similar histological features may exhibit markedly different biological behavior and clinical outcomes [4,7]. These findings underscore the need for additional, accessible tissue-based molecular markers that may complement routine diagnostic neuropathology.

Aquaporin-4 (AQP4) is a membrane water channel involved in brain water homeostasis, blood–brain barrier function, and regulation of extracellular fluid dynamics [8,9,10]. In intra-axial tumors such as gliomas, altered AQP4 expression and distribution have been associated with peritumoral edema, vascular permeability, and tumor invasiveness [10,11]. However, meningiomas arise from arachnoid cap cells and exhibit distinct stromal and vascular characteristics compared with glial tumors. Accordingly, the expression pattern and potential relevance of AQP4 in meningiomas remain insufficiently characterized. Previous studies have investigated the role of AQP4 in meningiomas, particularly in relation to peritumoral edema and tumor-associated vascular characteristics. AQP4 expression has been associated with fluid regulation and vascular permeability in intracranial tumors, including meningiomas [5,8,9]. However, findings specific to meningiomas remain limited and sometimes inconsistent, and the clinical relevance of AQP4 expression has not yet been fully clarified.

Non-coding RNAs, including microRNAs and long non-coding RNAs, are key regulators of gene expression and participate in a wide range of oncogenic and tumor-suppressive processes [12,13,14]. In meningiomas, several microRNAs have been associated with tumor proliferation, migration, and angiogenesis, and their dysregulation has been linked to higher WHO grade and more aggressive tumor behavior [15,16,17]. In addition, expression profiling studies suggest that microRNA signatures may provide complementary diagnostic or prognostic information [18].

Experimental studies in gliomas have suggested that certain microRNAs, including miR-216a and miR-320a, may be associated with pathways involving AQP4 expression and cellular motility [19,20]. Furthermore, the LINC00461/miR-216a/AQP4 pathway has been implicated in glioma biology [9]. However, given the distinct cellular origin and microenvironment of meningiomas, it remains unclear whether similar expression patterns or associations are present in these tumors.

miR-320a and miR-216a have been reported to directly target AQP4 by binding to its 3′ untranslated region, while LINC00461 may act as a competing endogenous RNA by sequestering miR-216a and modulating its regulatory effects. These interactions suggest a potential regulatory network involving AQP4 and non-coding RNAs, although their relevance in meningiomas has not been fully elucidated.

Based on these considerations, the present study aimed to evaluate the tissue-based expression patterns of AQP4 and selected non-coding RNAs (miR-216a, miR-320a, and LINC00461) in meningiomas across different histological grades. Rather than testing a specific mechanistic hypothesis, we sought to explore whether combined expression patterns at the tissue level may provide additional insight into the molecular heterogeneity of meningiomas beyond conventional histopathological classification.

## 2. Materials and Methods

### 2.1. Ethics Statement

This study was conducted in accordance with the Declaration of Helsinki. Ethical approval was obtained from the Institutional Review Board of Eskisehir Osmangazi University (Approval No: 2024-35). Written informed consent was obtained from all participants prior to tissue collection and analysis.

### 2.2. Study Population

A total of 78 individuals were included in this study. The patient group consisted of 65 individuals who underwent surgical resection for intracranial meningioma. Thirteen individuals without a history of intracranial neoplasia, who underwent decompressive or vascular neurosurgical procedures, served as non-neoplastic controls. Meningiomas were classified according to the 2021 World Health Organization (WHO) classification. Among the cases, 37 tumors were classified as WHO grade 1 and 28 tumors as WHO grade 2–3 [6]. Demographic and clinicopathological data were retrieved from institutional medical records.

### 2.3. Immunohistochemistry

Formalin-fixed, paraffin-embedded tissue blocks were sectioned at 4 µm thickness. Sections were deparaffinized in xylene and rehydrated through graded ethanol solutions. Antigen retrieval was performed using heat-induced epitope retrieval in citrate buffer (pH 6.0). After blocking endogenous peroxidase activity with hydrogen peroxide, sections were incubated with a primary antibody against aquaporin-4 (AQP4) according to the manufacturer’s protocol. A secondary antibody was subsequently applied, and immunoreactivity was visualized using diaminobenzidine as the chromogen. Slides were counterstained with hematoxylin. AQP4 expression was evaluated semi-quantitatively based on staining intensity and the proportion of positive cells. Scoring was performed independently by two experienced pathologists blinded to clinical data. Interobserver agreement was assessed using Cohen’s kappa coefficient (κ = 0.86).

### 2.4. RNA Isolation and Quantitative Reverse Transcription PCR

Total RNA, including small RNA fractions, was extracted from formalin-fixed paraffin-embedded tissue samples using the miRNeasy FFPE Kit (Qiagen, Hilden, Germany) according to the manufacturer’s instructions. cDNA synthesis for AQP4, LINC00461, and β-actin was performed using the VitaScript™ First-Strand cDNA Synthesis Kit (Procomcure Biotech GmbH, Bergheim, Austria). cDNA synthesis for miR-320a, miR-216a, and U6 was carried out using the miRNA All-In-One cDNA Synthesis Kit (Applied Biological Materials Inc., Richmond, BC, Canada). Real-time PCR was performed on an Applied Biosystems 7300 Real-Time PCR System using 2X Magic SYBR Mix (Procomcure Biotech GmbH, Bergheim, Austria) for mRNA/lncRNA targets and Blastag 2X qPCR Master Mix Kit (Applied Biological Materials Inc., Richmond, BC, Canada) for microRNA targets. Relative expression levels were calculated using the 2^−ΔΔCt^ method [21].

The primer sequences used for RT-PCR analysis were as follows: β-actin forward, GCCAACTTGTCCTTACCCAGA; β-actin reverse, AGGAAGAGAGACTTGACCC; LINC00461 forward, GGAATCTTAAGCGCGGCAAG; LINC00461 reverse, TTCATTCTCACACGCTCCCC; AQP4 forward, ACGCACACCTTGTTTTAATGCT; and AQP4 reverse, TCCAAACTGTCCCTAGAAAGGAA. Commercial microRNA primers were obtained from ABM Kit (Applied Biological Materials Inc., Richmond, BC, Canada): U6 (catalog no. MPH00001), miR-320a (catalog no. MPH01422), and miR-216a (catalog no. MPH01271).

### 2.5. Rationale for microRNA Selection

A candidate-based, hypothesis-driven approach was adopted due to the exploratory design and relatively limited cohort size. miR-216a and miR-320a were selected based on previous experimental studies suggesting potential associations with AQP4-related pathways in glioma models. LINC00461 was included as a representative long non-coding RNA implicated in microRNA-mediated regulatory processes [9,19,20]. This targeted approach enabled focused evaluation of biologically plausible molecular interactions at the tissue level.

### 2.6. Statistical Analysis

Statistical analyses were performed using IBM SPSS Statistics software (version 25, IBM Corp., Armonk, NY, USA). Continuous variables were expressed as mean ± standard deviation or median (range), depending on data distribution. Normality was assessed using the Kolmogorov–Smirnov test. Comparisons between two groups were performed using the Student’s *t*-test or Mann–Whitney U test, as appropriate. Comparisons among multiple groups were conducted using one-way ANOVA or the Kruskal–Wallis test. Categorical variables were analyzed using the chi-square test. Correlations between variables were assessed using Spearman’s rank correlation coefficient. Receiver operating characteristic (ROC) curve analysis was performed to evaluate diagnostic performance. A two-sided *p*-value < 0.05 was considered statistically significant.

## 3. Results

### 3.1. Patient Characteristics

A total of 65 patients with intracranial meningioma and 13 non-neoplastic control subjects were included. According to the 2021 World Health Organization (WHO) classification, 37 tumors were classified as grade 1 and 28 tumors as grade 2–3. The median age was 58 (34–86) years in the control group, 60 (36–84) years in the low-grade group, and 60 (4–86) years in the high-grade group, with no statistically significant difference between groups (*p* = 0.87). Demographic characteristics are summarized in Table 1.

### 3.2. Molecular Biomarker Expression

**Figure 1:**
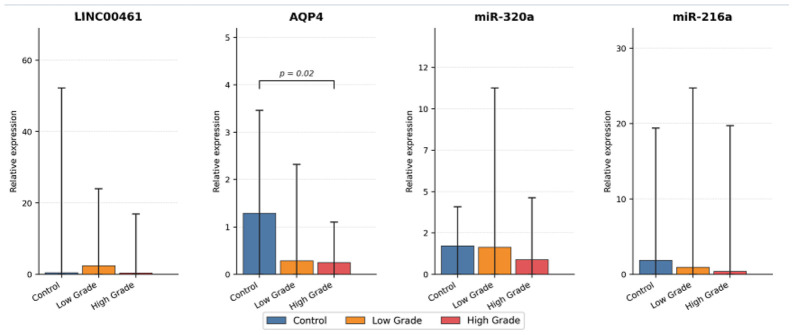
Expression patterns of molecular biomarkers across study groups. Relative expression levels of LINC00461, AQP4, miR-320a, and miR-216a in control, low-grade, and high-grade meningioma groups. Error bars represent standard deviation. AQP4 expression differed significantly between groups (*p* = 0.02), whereas other biomarkers did not reach statistical significance. These findings are illustrated in this figure, where error bars represent standard deviation and statistically significant differences are indicated (*p* < 0.05).

Expression levels of AQP4, LINC00461, miR-216a, and miR-320a were compared across control, low-grade, and high-grade groups (Table 2, Figure 1). Among the evaluated biomarkers, only AQP4 expression demonstrated a statistically significant difference between groups (*p* = 0.02). AQP4 levels were highest in the control group and decreased progressively in low- and high-grade tumors. Although miR-216a and miR-320a expression levels were lower in tumor groups compared with controls, these differences did not reach statistical significance (*p* = 0.32 and *p* = 0.25, respectively). LINC00461 expression showed variability across groups without statistically significant differences (*p* = 0.41). Overall, AQP4 represented the most consistently altered biomarker, whereas microRNA expression patterns were more variable and did not demonstrate clear group separation, possibly reflecting biological heterogeneity and limited statistical power.

### 3.3. Immunohistochemical Findings

**Figure 2:**
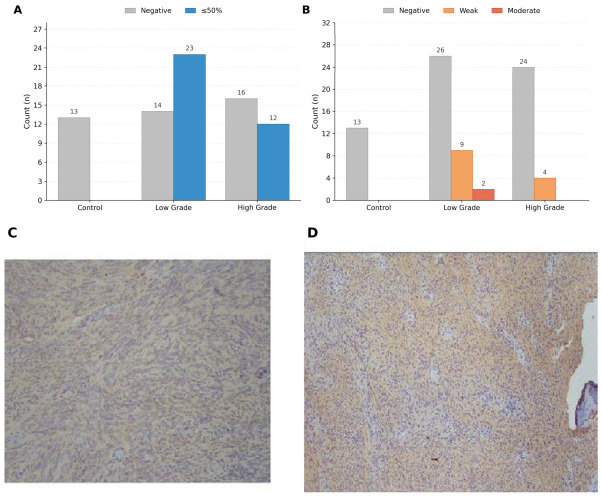
Immunohistochemical and correlation analyses of AQP4 expression. (**A**) Distribution of AQP4 staining proportions (negative, ≤50%, >50%) across control, low-grade, and high-grade groups. (**B**) Distribution of AQP4 staining intensity (negative, weak, moderate, strong) across groups. (**C**) Representative microphotograph of AQP4 staining in control tissue. (**D**) Representative microphotograph of AQP4 staining in meningioma tissue. Significant differences in staining patterns were observed between control and tumor groups (*p* < 0.001).

Immunohistochemical analysis revealed significant differences in both staining proportion and staining intensity between control and tumor groups (Figure 2A,B). All control samples were negative for AQP4 staining, whereas meningioma samples demonstrated variable expression. In tumor tissues, staining was predominantly weak to moderate, with distribution across both ≤50% and >50% categories. These differences were statistically significant (*p* < 0.001), indicating distinct immunohistochemical profiles between non-neoplastic and tumor tissues.

### 3.4. Comparison Between Patient and Control Groups

When all meningioma cases (*n* = 65) were compared with controls (*n* = 13), AQP4, miR-216a, and miR-320a expression levels were higher in controls, whereas LINC00461 levels were higher in tumor samples.

However, only AQP4 expression demonstrated a statistically significant difference between groups (*p* = 0.006; Table 3).

### 3.5. Correlation Analysis

**Figure 3:**
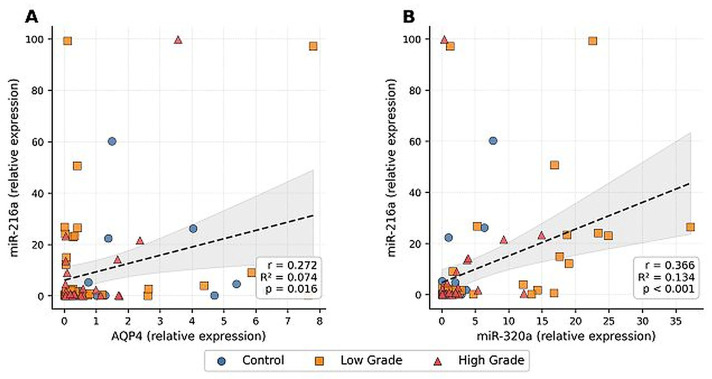
Correlation scatter plots. Figure 3. Correlation scatter plots. (**A**) Correlation between AQP4 and miR-216a expression. (**B**) Correlation between miR-320a and miR-216a expression.

Correlation analysis was performed to evaluate relationships between molecular biomarkers (Table 4). AQP4 expression showed a modest but statistically significant positive correlation with miR-216a (r = 0.272, *p* = 0.016). In addition, miR-216a expression was positively correlated with miR-320a (r = 0.366, *p* = 0.001). No statistically significant correlations were observed among other biomarkers, suggesting limited but detectable associations within the study cohort, likely reflecting indirect or context-dependent relationships (Figure 3).

### 3.6. ROC Analysis

**Figure 4:**
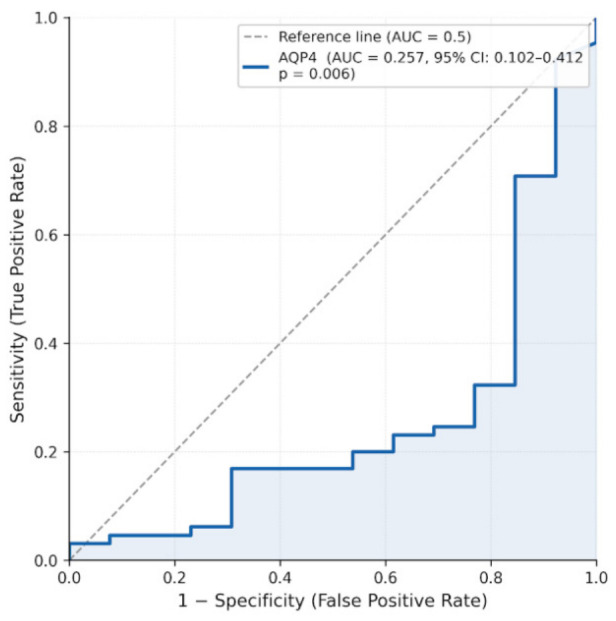
Receiver operating characteristic (ROC) curve for AQP4 expression in distinguishing meningioma from control samples. The area under the curve (AUC) was 0.257 (95% CI: 0.102–0.412, *p* = 0.006), indicating an inverse association between AQP4 expression and tumor presence.

Receiver operating characteristic (ROC) analysis demonstrated a statistically significant discriminatory performance for AQP4 (AUC = 0.257, 95% CI: 0.102–0.412, *p* = 0.006, Figure 4). Given that AQP4 expression was reduced in tumor samples, the inverse AUC value reflects a negative association, indicating that lower AQP4 levels are associated with the presence of meningioma rather than higher values and when directionality is considered, this corresponds to moderate discriminatory ability. Overall, these findings suggest limited standalone diagnostic performance, while supporting a consistent relationship between reduced AQP4 expression and tumor status.

## 4. Discussion

This study demonstrates that Aquaporin-4 (AQP4) expression is significantly reduced in meningiomas compared with non-neoplastic control tissues and shows a further decrease in higher histological grades. These findings suggest that AQP4 expression in meningiomas follows a pattern distinct from intra-axial tumors such as gliomas, in which AQP4 is often upregulated and redistributed in association with edema and infiltrative growth [10,11]. The extra-axial origin and unique stromal architecture of meningiomas may therefore influence AQP4 expression in a tumor-specific manner.

The 2021 WHO classification emphasizes the integration of molecular features into tumor diagnosis and grading to improve prognostic accuracy beyond conventional histopathology [5,6]. Molecularly integrated grading systems and DNA methylation-based classifications have shown that tumors with similar histological characteristics may display markedly different biological behavior [3,4]. Within this context, the progressive reduction of AQP4 expression observed in our cohort may represent one component of the broader molecular heterogeneity that is not fully captured by routine histological evaluation.

In gliomas, AQP4 has been associated with blood–brain barrier function, peritumoral edema, and extracellular fluid dynamics [8,9,10]. Although meningiomas lack astrocytic lineage, their vascular organization and extracellular matrix composition may still be influenced by water channel regulation. The observed downregulation of AQP4 in higher-grade tumors may therefore reflect alterations in tumor microenvironment organization rather than mechanisms directly comparable to intra-axial tumors. This finding should be interpreted alongside the discrepancy observed between mRNA expression and immunohistochemical results.

Non-coding RNAs are important regulators of gene expression and have been implicated in multiple aspects of tumor biology [12,13,14]. In meningiomas, several microRNAs have been associated with tumor progression and aggressiveness [15,16,17]. In the present study, miR-216a and miR-320a levels were lower in tumor samples compared with controls; however, these differences did not reach statistical significance across histological grades. This finding likely reflects the limited statistical power of subgroup analyses and suggests that microRNA alterations in this context may be more subtle or heterogeneous. This may reflect limited statistical power rather than absence of a biological effect.

Previous studies have demonstrated that miR-320a and miR-216a can directly target AQP4 by binding to its 3′ untranslated region, thereby modulating its expression [18,19,20]. In addition, LINC00461 has been proposed to function as a competing endogenous RNA (ceRNA), acting as a molecular sponge that sequesters miR-216a and prevents it from interacting with AQP4 [18]. These findings suggest the presence of a regulatory network involving AQP4 and non-coding RNAs. In this context, the correlations observed in our study may reflect indirect or context-dependent regulatory interactions rather than direct causality, particularly considering the distinct biological microenvironment of meningiomas compared with gliomas. However, the present study was not designed to evaluate direct molecular interactions, and therefore these findings should be interpreted as associative rather than mechanistic.

The observed correlations between AQP4 and miR-216a, as well as between miR-216a and miR-320a, indicate the presence of coordinated expression patterns within the dataset. Previous experimental studies in gliomas have suggested potential interactions between these molecules [9,19,20]. However, given the distinct biological context of meningiomas, these associations should be interpreted cautiously. Rather than indicating a direct regulatory mechanism, the correlations observed in this study may reflect context-dependent or indirect relationships within tumor tissue.

From a diagnostic perspective, ROC analysis demonstrated statistically significant but limited discriminatory performance for AQP4. The inverse AUC value is consistent with reduced AQP4 expression in tumor tissues, indicating that lower expression levels are associated with disease presence. However, the overall performance suggests that AQP4 alone is insufficient as a standalone diagnostic marker. This aligns with the broader understanding that single biomarkers rarely capture the complexity of tumor biology, and that combined molecular approaches may provide more informative insights. When directionality is considered, this corresponds to moderate discriminatory ability.

In this context, the combined assessment of AQP4 and selected microRNAs may offer supplementary information regarding tumor heterogeneity at the tissue level. Although individual markers did not demonstrate strong diagnostic performance, their collective patterns may reflect underlying biological variability not fully captured by histopathological classification.

This study was designed as a targeted, exploratory analysis focusing on a limited number of biologically plausible candidates rather than a comprehensive molecular profiling approach. The selection of miR-216a, miR-320a, and LINC00461 was based on prior experimental data, allowing focused evaluation within a single-center cohort. Accordingly, the findings should be considered hypothesis-generating.

Several limitations should be acknowledged. The retrospective design and relatively small sample size limit generalizability and reduce statistical power, particularly for subgroup comparisons. Functional validation experiments were not performed, and therefore, causal or regulatory relationships cannot be established. In addition, control tissues were obtained from patients undergoing neurosurgical procedures, which may not fully represent normal meningeal tissue. However, this approach is consistent with prior studies in neurosurgical molecular pathology where access to normal tissue is limited. In addition, no formal correction for multiple comparisons was applied, and therefore, the results should be interpreted with caution.

Despite these limitations, the combined use of immunohistochemistry and quantitative PCR provides integrated, tissue-based molecular data that may be relevant for routine diagnostic practice. These findings contribute to the growing body of evidence supporting the role of supplementary molecular markers in characterizing meningioma heterogeneity.

## 5. Conclusions

AQP4 expression is consistently reduced in meningiomas compared with non-neoplastic tissues and shows a further decrease in higher histological grades. In contrast, associated microRNA alterations demonstrate variable expression patterns without clear group separation.

These findings support the presence of molecular heterogeneity among meningiomas beyond conventional histopathological classification. While AQP4 alone shows limited diagnostic performance, its combined evaluation with selected microRNAs may provide complementary tissue-level information when interpreted alongside standard pathology.

Overall, these results are exploratory and highlight the need for larger, multicenter studies and functional investigations to clarify the biological and potential clinical relevance of these molecular patterns in meningiomas. These findings should be considered exploratory.

## Figures and Tables

**Table 1:** Demographic and clinicopathological characteristics of the study population.

Variable	Control (*n* = 13)	Low-Grade (*n* = 37)	High-Grade (*n* = 28)	*p*-Value
Age (years), median (range)	58 (34–86)	60 (36–84)	60 (4–86)	0.87

*p*-value represents comparison of age between groups (Kruskal–Wallis test).

**Table 2:** Expression levels of AQP4, LINC00461, miR-216a, and miR-320a across study groups.

Biomarker	Control Median (Range)	Low-Grade Median (Range)	High-Grade Median (Range)	*p*-Value
LINC00461	0.40 (0.01–187.60)	2.41 (0.01–93.15)	0.36 (0.00–79.43)	0.41
AQP4	1.29 (0.03–6.34)	0.28 (0.01–7.80)	0.25 (0.04–3.56)	**0.02**
miR-320a	1.71 (0.03–7.66)	1.63 (0.07–37.21)	0.88 (0.05–14.90)	0.25
miR-216a	1.84 (0.01–60.19)	0.92 (0.01–99.15)	0.39 (0.00–99.84)	0.32

Values expressed as median (range). *p*-values derived from the Kruskal–Wallis test. Bold indicates statistical significance.

**Table 3:** Comparison of biomarker expression between control and meningioma groups.

Biomarker	Control Median (Range)	Meningioma Median (Range)	Z	*p*-Value
LINC00461	0.40 (0.01–187.60)	1.35 (0.00–93.15)	0.40	0.687
AQP4	1.29 (0.03–6.34)	0.27 (0.01–7.80)	2.75	**0.006**
miR-320a	1.71 (0.03–7.66)	1.27 (0.05–37.21)	0.26	0.794
miR-216a	1.84 (0.01–60.19)	0.79 (0.00–99.84)	0.40	0.683

Values expressed as median (range). *p*-values derived from the Mann–Whitney U test. Bold indicates statistical significance.

**Table 4:** Correlation analysis between molecular biomarkers.

	LINC00461	AQP4	miR-320a	miR-216a
LINC00461	**–**	r = 0.203, *p* = 0.074	r = −0.124, *p* = 0.278	r = −0.048, *p* = 0.678
AQP4	r = 0.203, *p* = 0.074	–	r = −0.105, *p* = 0.360	**r = 0.272, *p* = 0.016**
miR-320a	r = −0.124, *p* = 0.278	r = −0.105, *p* = 0.360	–	**r = 0.366, *p* = 0.001**
miR-216a	r = −0.048, *p* = 0.678	**r = 0.272, *p* = 0.016**	**r = 0.366, *p* = 0.001**	**–**

Spearman correlation coefficients (r) and corresponding *p*-values. Bold values indicate statistically significant correlations.

## Data Availability

The datasets generated and/or analyzed during the current study are available from the corresponding author upon request.
